# Investigating middle school students' creative problem solving in numerical and spatial domains

**DOI:** 10.3389/fpsyg.2025.1686498

**Published:** 2025-11-04

**Authors:** Semahat Incikabi

**Affiliations:** Department of Mathematics Education, Faculty of Education, Sinop University, Sinop, Türkiye

**Keywords:** students' creative products, creative problem solving, numeracy skills, spatial skills, mathematical creativity

## Abstract

**Objective:**

This study aimed to investigate the relationship between creativity components in numerical and spatial mathematical problem-solving contexts and to identify the characteristics of products generated by students with different levels of creativity.

**Methods:**

The study involved 167 sixth-grade students (aged 12–13) from eight public schools in Turkey. Data were collected using the Divergent Production Ability in Mathematical Problem Solving Test (DPAMPS). Students' responses were evaluated using a rubric adapted from established creativity frameworks, and statistical analyses were conducted to examine relationships between creativity constructs and to classify students into high and low creativity groups.

**Results:**

Findings revealed no statistically significant correlation between students' spatial and numerical creativity scores, suggesting that these domains function independently. Regardless of creativity level, most students produced prototypical responses, such as right triangles in spatial tasks and parity or divisibility in numerical tasks, indicating reliance on conventional representations. However, students with high creative ability demonstrated greater fluency and flexibility, generating more diverse and atypical solutions across both domains.

**Discussion:**

The results support the domain-specific nature of creativity in mathematical contexts and highlight how curricular and instructional practices may limit opportunities for students to express originality. Even high-ability students tended to reproduce familiar patterns, reflecting prototype-driven reasoning reinforced by curricular settings. The study underscores the need for open-ended, non-routine mathematical tasks that encourage divergent thinking and integration of spatial and numerical reasoning to better cultivate students' mathematical creativity.

## Introduction

In recent years, research and interest in creativity have increased significantly, with a clear shift in the focus of studies from general (interdisciplinary) creativity to domain-specific (specialized) creativity. This transformation reflects a growing awareness that creativity manifests itself in different ways across different fields and emphasizes the need to understand how creative processes operate in specific domains such as art, science, and technology. Researchers such as Plucker and Zabelina (2009) have highlighted the importance of viewing creativity not as a uniform, all-encompassing trait, but as a nuanced skill that can develop context-dependently and be expressed in different ways. This shift in perspective has encouraged goal-oriented research aimed at understanding how creative skills develop and function in specific areas of expertise, thereby seeking to provide a deeper understanding of how creativity can be developed in specific contexts and utilized in innovation.

The domain specificity hypothesis proposes that creativity manifests itself in different ways across disciplines and contexts and is shaped by the unique cognitive demands of each domain (Baer, 1998). Mathematical creativity is generally defined as the ability to generate original, functional, and insightful solutions to mathematical problems (Sternberg and Lubart, 1998). In the field of mathematics education, the development of problem-solving skills is widely accepted as a critical component in supporting mathematical creativity (Haylock, 1985; Silver, 1997). Students who demonstrate high levels of mathematical creativity have been observed to produce a wider range of innovative solutions compared to their peers (e.g., Chamberlin and Moon, 2005). The interaction between spatial and numerical creativity is particularly evident in interdisciplinary mathematical problem-solving contexts. For example, the process of graphing equations combines numerical accuracy with spatial visualization and requires students to make a seamless transition between symbolic representations and visual interpretations. Similarly, solving real-life optimization problems, such as designing structures that minimize material usage, requires the integration of spatial reasoning to conceptualize shapes and numerical creativity to calculate dimensions and costs. Such interactions demonstrate that spatial and numerical creativity function not as independent but as complementary skills in holistic problem-solving processes (Wai et al., 2009).

Mathematical creativity encompasses skills such as performing operations with numbers, quantitative reasoning, and understanding algebraic structures. This type of creativity is particularly evident in activities that require innovative problem-solving within a symbolic framework, such as developing new formulas or optimizing solutions. In contrast, spatial creativity relies on the ability to perceive objects in space and manipulate them mentally; this is fundamental for tasks such as constructing geometric proofs, interpreting diagrams, and three-dimensional modeling (Cheng and Mix, 2014). Although these domains have different characteristics, their interaction is becoming increasingly important in the context of mathematics education, where challenges requiring the integration of numerical accuracy and spatial intuition are frequently encountered (Wai et al., 2009). This study examines how students use and integrate their numerical and spatial skills when solving complex problems. Numerical and spatial skills are fundamental building blocks of mathematical reasoning and problem solving; developing these skills can broadly affect students' overall academic and cognitive development (Amado et al., 2018). Furthermore, theories explaining the close interaction between creativity and numerical and visual/spatial intelligence have also been developed (Clark and Zimmerman, 1997; Gardner, 1988a,b; Torrance, 1966, 1988).

It is my intention to examine the relationship between the spatial and numerical components of creativity in mathematical problem solving to determine the most appropriate sequence and structure of tasks to be presented to students. Furthermore, such a study could contribute to the existing literature by clarifying the relationship between numerical problem solving and spatial problem solving in the context of creativity. In addition, examining the characteristics of the products produced by students with different levels of creativity within the scope of this study will provide valuable insights into the reflection of creativity in student products. Therefore, this research aims to reveal the relationship between creativity components and tasks defined in numerical and spatial domains and to examine the characteristics of products produced by students with different levels of creativity in various domains. In this context, the research question is defined as follows:

1) Is there a relationship between the constructs of creative skills in mathematical problem-solving in spatial and numerical domains?
2) What are the characteristics of the products produced by students with different levels of mathematical creativity in problems involving numerical and spatial domains?

### The connection between creativity and problem solving in mathematics

Mathematical creativity is generally accepted as an integral component of mathematical ability and is mostly studied through problem-solving and problem-posing activities (Silver, 1997). While some researchers view creativity as an interdisciplinary trait and conceptualize it as a universal skill (Guilford, 1959), others argue that creativity is domain-specific and requires specialized knowledge in a particular field, such as mathematics (Balka, 1974; Silver, 1997; Van Harpen and Sriraman, 2013). Different definitions of mathematical creativity emphasize various dimensions. These definitions include the ability to generate original solutions (Sriraman, 2009), the capacity for flexible thinking (Haylock, 1997), and the ability to develop innovative problem-solving techniques (Haylock, 1987). Within the scope of this study, mathematical creativity is defined as the ability to identify and apply acceptable mathematical patterns and models to produce new mathematical ideas or products (Bicer, 2021).

Guilford (1959) model of general intelligence has four basic components of divergent thinking: fluency, flexibility, originality, and elaboration. These components have been widely adapted to assess mathematical creativity (Balka, 1974; Bicer et al., 2020). Fluency refers to the ability to generate multiple solutions to a given problem; flexibility refers to the ability to propose different and varied solution paths; and originality refers to the degree to which the generated solutions are unique compared to others (Silver, 1997). This study used these dimensions to examine the relationship between creative abilities in mathematical problem-solving processes.

Numerous studies support a strong relationship between mathematical creativity and problem solving (Lee and Hoffman, 2014; Titus and Koppitsch, 2018; Valentine et al., 2017). Some researchers argue that mathematical creativity is essentially a form of problem solving (Haylock, 1985; Kim et al., 2019). For example, Tyagi (2016) found a significant relationship between students' levels of mathematical creativity and their performance on problem-solving tasks. Similarly, Lin and Cho (2011) found that mathematical creativity directly predicts problem-solving ability. However, some studies suggest that problem-solving performance may be a precursor to creativity rather than creativity itself (Khalid et al., 2020). In this study, creative problem solving is the application of creativity to develop innovative and effective solutions to structured mathematical problems (Khalid et al., 2020; Kim et al., 2003).

### Creativity in the domains of numeracy and spatial skills

Research in the field of creativity argues that creativity is a complex and multidimensional process that requires the convergence of various talents, skills, and cognitive resources. According to some researchers, creativity emerges from everyday cognitive processes accessible to everyone, such as memory, problem solving, imagination, and analogical reasoning (Chrysikou, 2019). Haylock (1985), on the other hand, argued that creativity assessments should be included in school mathematics curricula, emphasizing the importance of integrating numerical and spatial problem-solving tasks into measurement frameworks to encourage and assess creativity in mathematical contexts.

Spatial skills involve the ability to visualize and manipulate objects mentally and are of fundamental importance in disciplines such as mathematics, engineering, and art (Lohman, 1996). Creativity and spatial skills are closely related cognitive structures that significantly contribute to problem-solving and artistic expression processes. Research shows that individuals with high spatial skills perform superiorly on tasks requiring mental transformation of objects, which plays a critical role in creative problem-solving processes (Shepard and Metzler, 1971; Uttal et al., 2013). For example, architects and engineers design and develop innovative structures using spatial visualization, demonstrating the strong synergy between creativity and spatial reasoning (Sorby, 2009). Research also reveals a meaningful relationship between visual perception and creative production. Various studies in psychology and neuroscience have examined the role of visual-spatial abilities in creativity and innovation processes (Humphreys et al., 1993; Kell et al., 2013; Liu, 2007). Neuroscience research has revealed that when individuals engage in tasks requiring the use of objects' perceptual properties (e.g., using a tennis racket as a snow boot), there is increased activity in the middle occipital gyrus, a brain region associated with visual perception (Chrysikou and Thompson-Schill, 2011). This finding suggests that tasks requiring creative production through perceptual processing are linked to visual cognitive functions. Furthermore, fluid intelligence, defined as the capacity to solve new problems through visual-spatial and verbal reasoning, has been found to play a key role in performing various creative tasks (Beaty et al., 2019).

Numeracy is the ability to apply quantitative reasoning in daily life and is considered a fundamental competency for academic success. This skill encompasses the ability to perform calculations, interpret numerical data, and apply it to real-life contexts (Millett et al., 2004; Steen, 2002). (Grégoire 2016) states that numeracy forms the basis of various cognitive processes, including critical and creative thinking, and is central to higher-level problem-solving skills. This perspective emphasizes that numeracy is not limited to basic calculation skills but is also an integral part of creative problem solving; this skill plays a critical role, especially in areas where quantitative reasoning is necessary (Pugalee, 1999; Purnomo et al., 2023). Numeracy skills are important for students when solving practical mathematical problems. In addition, they help individuals develop sensitivity to data, patterns, and numerical relationships, strengthening their reasoning skills (Lee-Post, 2019; Yana et al., 2024).

The researchers have extensively studied the relationship between creativity and numeracy skills. Positive student attitudes toward mathematics have been shown to encourage higher participation and improved performance in numerical tasks (Hannula, 2002; Di Martino and Zan, 2010). Research reveals that students with high levels of creative thinking skills approach mathematical problems more innovatively, generate multiple solutions, and demonstrate greater flexibility in their thinking processes (Leikin, 2009; Runco and Jaeger, 2012). In addition, cognitive styles such as domain independence also influence the relationship between creativity and numerical literacy. Domain-independent individuals demonstrate stronger creative problem-solving skills than their domain-dependent peers (Sternberg, 1997; Witkin et al., 1967). Furthermore, students‘ Adversity Quotient (AQ) has also been linked to numerical creativity, and resilience and motivation have been found to play a critical role in developing creativity and numerical skills (Martin and Marsh, 2008; Stoltz, 1999). These findings point to the complex and multidimensional nature of the relationship between creativity and numerical literacy, emphasizing the importance of supporting students' positive attitudes toward mathematics and using goal-directed motivational strategies to enhance student achievement.

## Methodology

### Context and participants

This section summarizes the problem-solving experiences of middle school students in Turkey. Problem solving was first recognized as a curricular skill in the mathematics teaching program in 1949, emphasizing that students should “approach problems and situations in all areas of life quantitatively” (Ministry of National Education, 1949, p. 127). The curriculum, which was renewed in 2024, continues this emphasis and defines problem solving as a fundamental skill specific to areas that include analysis, interpretation, mathematical solution development, and reflection processes (Ministry of National Education, 2024, p. 15). These competencies have been integrated into all mathematical content areas.

Mathematics textbooks developed in line with the objectives of Turkey's national mathematics teaching program are used as basic teaching tools. The content of the books directly shapes classroom practices. Including problem-solving tasks in textbooks reflects the priority of this skill in the curriculum and significantly affects students' skill development (Ev-Çimen and Yıldız, 2017). In line with these textbooks, which are mandatory by the Ministry of National Education, teachers generally follow the prescribed content and pedagogical approaches (Ulusoy and Incikabi, 2020, 2023). Therefore, the problem-solving tasks encountered by Turkish students largely stem from curriculum guidelines and textbook content (Arikan and Ünal, 2015).

Considering the homogeneous structure of classrooms, an appropriate sampling method was used to select the sample within the scope of Turkey's national education system. In this context, eight public schools that were similar in terms of student achievement and socioeconomic level were included in the study. Teachers facilitated voluntary participation, and 181 sixth-grade students participated in the study. Since complete data was needed for a reliable analysis due to missing responses, a list-based deletion method was applied, which reduced the number of participants. The final sample consists of 167 sixth-grade students (aged 12–13) studying in a coastal city in northern Turkey. The gender distribution is balanced, with 49% of participants being male and 51% female. The detailed distribution of participants by school is presented in Table 1.

**Table 1:** Sample of the study according to the schools.

**School ID**	**Boy**	**Girl**	**Total**
School-1	8	12	20
School-2	11	10	21
School-3	10	12	22
School-4	12	8	20
School-5	11	9	20
School-6	9	11	20
School-7	11	12	23
School-8	10	11	21
Total	82	85	167

### Instruments and procedures

The assessment of creativity has historically been based on divergent production tests. Such assessments typically use a three-parameter framework to measure creativity. This framework encompasses the dimensions of fluency (number of responses produced), flexibility (variety of response categories), and originality (rarity of responses). In this context, it has been previously demonstrated that open-ended problems and tasks with multiple solution paths, which include Torrance's (1966, 1988) three indicators of creativity, are frequently used (Sadak et al., 2022; Ulusoy et al., 2025). These approaches have been adapted to the field of mathematics, and the validity of mathematical creativity ability has been supported by various studies (e.g., Leikin, 2007; Levav-Waynberg and Leikin, 2012; Van Harpen and Sriraman, 2013). To achieve the objectives of this study, the Divergent Production Ability in Mathematical Problem Solving Test (DPAMPS) was administered to students to assess their divergent production skills in mathematical problem solving. According to Haylock (1984), the fundamental way to align creativity assessments with the school mathematics curriculum is to include both numerical and spatial contexts; this approach also reflects the emphasis placed on the number and geometry/measurement domains in the curriculum. In this vein, the DPAMPS, with its open-ended structure requiring different responses, consists of two problem-solving tasks adapted from Haylock (1984) to assess students' numerical and spatial abilities:

Numerical task: Compile a list of the common features shared by the numbers 16 and 36.

Spatial task: Draw polygons with an area of 2 *cm*^2^ on the dotted paper given below. Make sure that the polygons you draw are different. Horizontal and vertical distance between the dots is 1 cm.


$$\begin{array}{l} \;...\;...\\ \;...\;... \end{array}$$

Numerical task adopted in the DPAMPS involved problem-solving based on the concept of redefinition. Students were asked to examine two different numbers and identify ways in which they are similar. Spatial task was a problem-solving task with multiple possible solutions, where students needed to find as many different shapes as possible with an area of 2 cm^2^ by connecting dots on a nine-dot centimeter grid with straight lines. The tasks were translated into Turkish and evaluated for linguistic consistency by two language experts prior to their administration to the sample. Two experts, who have numerous studies in the field of mathematical creativity, verified the items placed in the DPAMPS. The internal consistency of the scores, as measured by Cronbach's alpha, was 0.88 for the DPAMPS, indicating a moderate to high level of reliability (Murphy and Davidshofer, 2001).

The DPAMPS was administered to students in a paper-based format, structured to facilitate the generation of diverse and varied solutions. To support this objective, students were encouraged to utilize additional sheets of paper as required. The allocated 40-min duration, corresponding to a standard class period, provided adequate time for students to demonstrate mathematical creativity, operationalized as fluency, flexibility, and originality, through engagement with problem-solving tasks.

While accepting the psychometric principle that longer tests produce more reliable scores by minimizing random measurement errors, this assessment was designed considering the students‘ developmental level (6th grade), time constraints in the school environment, and the cognitive demands of divergent thinking tasks. The use of a limited number of tasks for each domain is also consistent with previous research on assessing mathematical creativity in middle school students (e.g., Bicer et al., 2020; Haylock, 1987; Kar et al., 2019; Sadak et al., 2022). The primary goal in this study was to strike a balance between reliability and feasibility. Therefore, particular attention was paid to ensuring that students' tasks were open-ended and manageable. Furthermore, since the students participating in the study were not identified as gifted, expecting them to generate ideas for a large number of tasks could have negatively affected both their motivation to participate and the quality of the data collected.

### The scoring of DPAMPS test

Field experts first evaluated the solutions produced by students for the tasks in terms of appropriateness and validity. Responses inappropriate for the task were not included in subsequent evaluations. Solutions that did not sufficiently address the problem statement or were incomplete were also considered inappropriate responses. Appropriate and viable solutions were then scored according to fluency, flexibility, and originality, indicators of mathematical creativity. A rubric based on the interpretations of these indicators in the context of problem solving was developed (see Table 2), drawing on previous research (e.g., Sadak et al., 2022; Silver, 1997; Van Harpen and Sriraman, 2013; Yuan and Sriraman, 2011). The content validity of the rubric was verified by expert mathematics educators who have academic work in mathematical creativity and problem solving, but did not participate in this research.

**Table 2:** Rubric for assessing fluency and flexibility in problem solving (adapted from Sadak et al., 2022).

**Indicators**	**0**	**10**	**20**	**30**	**40**
Fluency	Solutions are not viable and appropriate	A student provided one viable and appropriate solution	A student provided two viable and appropriate solutions	A student provided three viable and appropriate solutions	A student provided more than three viable and appropriate solutions
Flexibility	Solutions are not viable and appropriate	A student implemented one approach to solve problem among all his/her solutions	A student implemented two approaches to solve problem among all his/her solutions	A student implemented three approaches to solve problem among all his/her solutions	A student implemented more than three approaches to solve problem among all his/her solutions

The number of correct solutions measured fluency to assess creative ability in problem solving. Flexibility was determined by the number of different solution types or categories produced by the students. The dimension of originality was assessed using a statistical rarity approach. In this method, responses were coded according to their frequency of occurrence within the sample; that is, responses given less frequently were assigned higher originality scores. This approach helps reduce subjective judgments in the evaluation and increases repeatability (Leikin et al., 2009; Silver, 1997). In assessing divergent thinking skills, frequency-based scoring is consistent with approaches that advocate for more objective measurement rather than methods based solely on evaluators' judgments (Plucker et al., 2011; Reiter-Palmon et al., 2019). Although alternative methods such as percentile scoring or fixed response constraints exist to account for the effect of fluency (Plucker et al., 2011), statistical rarity scoring is reliable in open-ended mathematical tasks and was therefore considered an appropriate method for the present study.

In the literature, there are different approaches to determining originality scoring ranges. For example, Sadak et al. (2022), Van Harpen and Sriraman (2013), and Yuan and Sriraman (2011) awarded the highest originality score (40 points) when the given responses were produced by 10% or fewer students. In contrast, Kattou et al. (2013) assigned the highest score only to responses given by 1% or fewer students. Furthermore, the percentage ranges used for intermediate scores may also differ across studies. In this study, if one or more of the student's responses were seen in less than 1% of all responses in the sample, 40 points were given; if seen between 1% and 10%, 30 points were given; if seen between 10% and 25%, 20 points were given; if seen between 26% and 40%, 10 points were given; and if seen in more than 40%, 0 points were given. Each student received three scores for each mathematical creativity task: fluency, flexibility, and originality. Finally, the students' DPAMPS total scores were calculated by taking the average of the scores obtained from all questions on the test.

**Figure 1:**
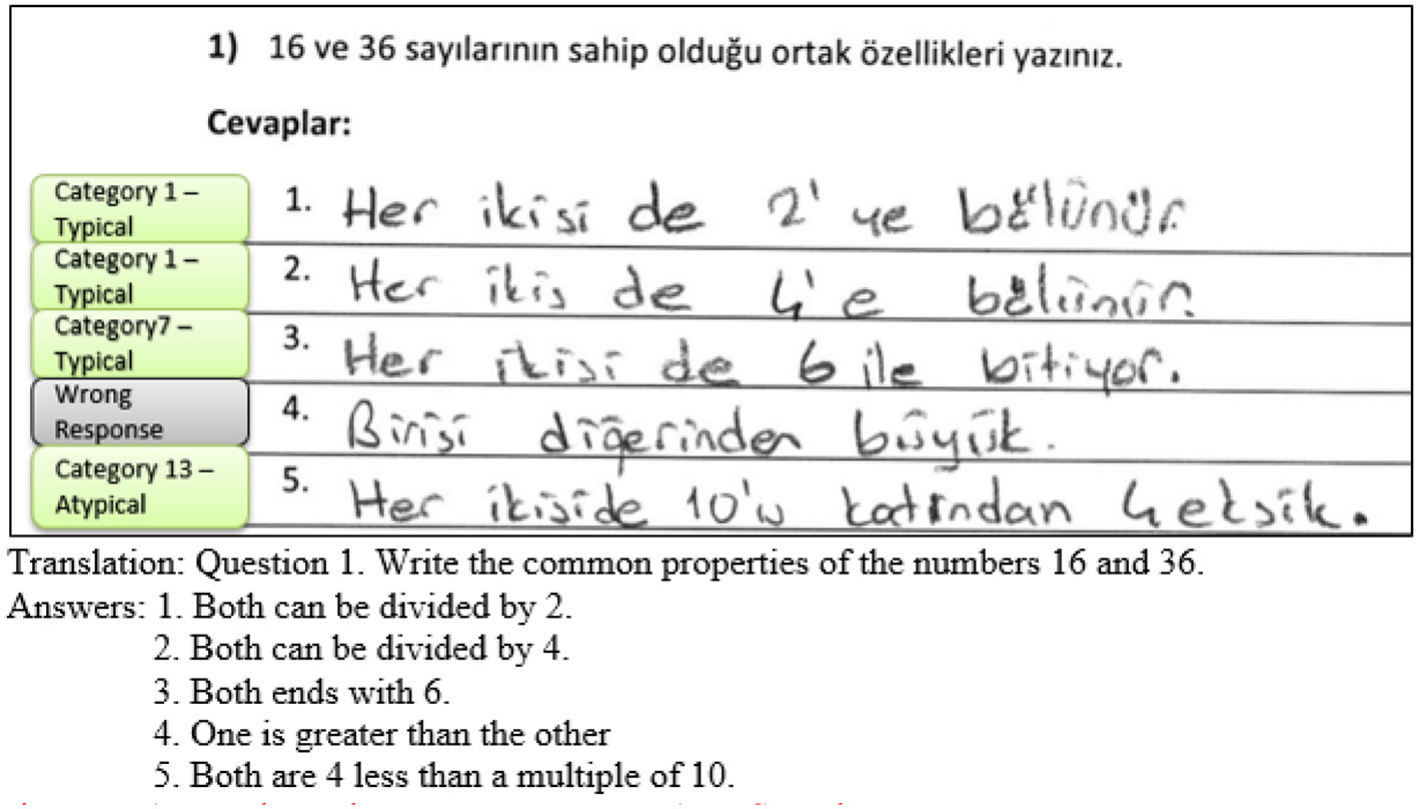
A sample student response to a DPAMPS-Task 1.

Figure 1 illustrates a student's response to the numerical task and explains the coding and scoring procedure. The responses of the DPAMPS instruments were analyzed based on the product categories established by Haylock (1984), with products not previously identified in Haylock's study categorized as new entries (see Appendix 1).

As illustrated in Figure 1, the student provided five explanations of the common properties of the numbers 16 and 36. However, only four of these properties could be deemed correct. Response 4, which compares the quantity of the numbers, is not a common property that both numbers have. Conversely, Responses 1 and 2 were categorized as “They are both multiples of… (or, both can be divided by…)” due to both responses stating numbers divisibility by 2 (response 1) and 4 (response 2). Moreover, response 3 was coded as “statements about the 6” category since the response states. Furthermore, the response 5 was coded in the category of “Arithmetic Relationships” since the statement included a common property of both numbers by using multiplication and subtraction operations. Ultimately, the student provided four correct responses in three distinct categories. As a result, she received 40 points for numerical fluency for generating four valid problems, regardless of whether they fell within the same or different categories. Additionally, she was awarded 30 points for numerical flexibility, as the three correct problems were coded into distinct categories. For her numerical originality score, she received 0 points for the responses 1 and 2 as more than 40% of her class mates (f = 167) had posed problems in the “They are both multiples of… (or, both can be divided by…)” category, 10 points for the response 3 as only 37% of students had posed a response in the “statements about the 6” category. Moreover, she earned 30 points for response 5, as 8% of her class mates (f = 167) had posed problems in the “Arithmetic Relationships” category. Consequently, her score for Numerical Originality was 30 points for the most original work (highest point that she earned).

**Figure 2:**
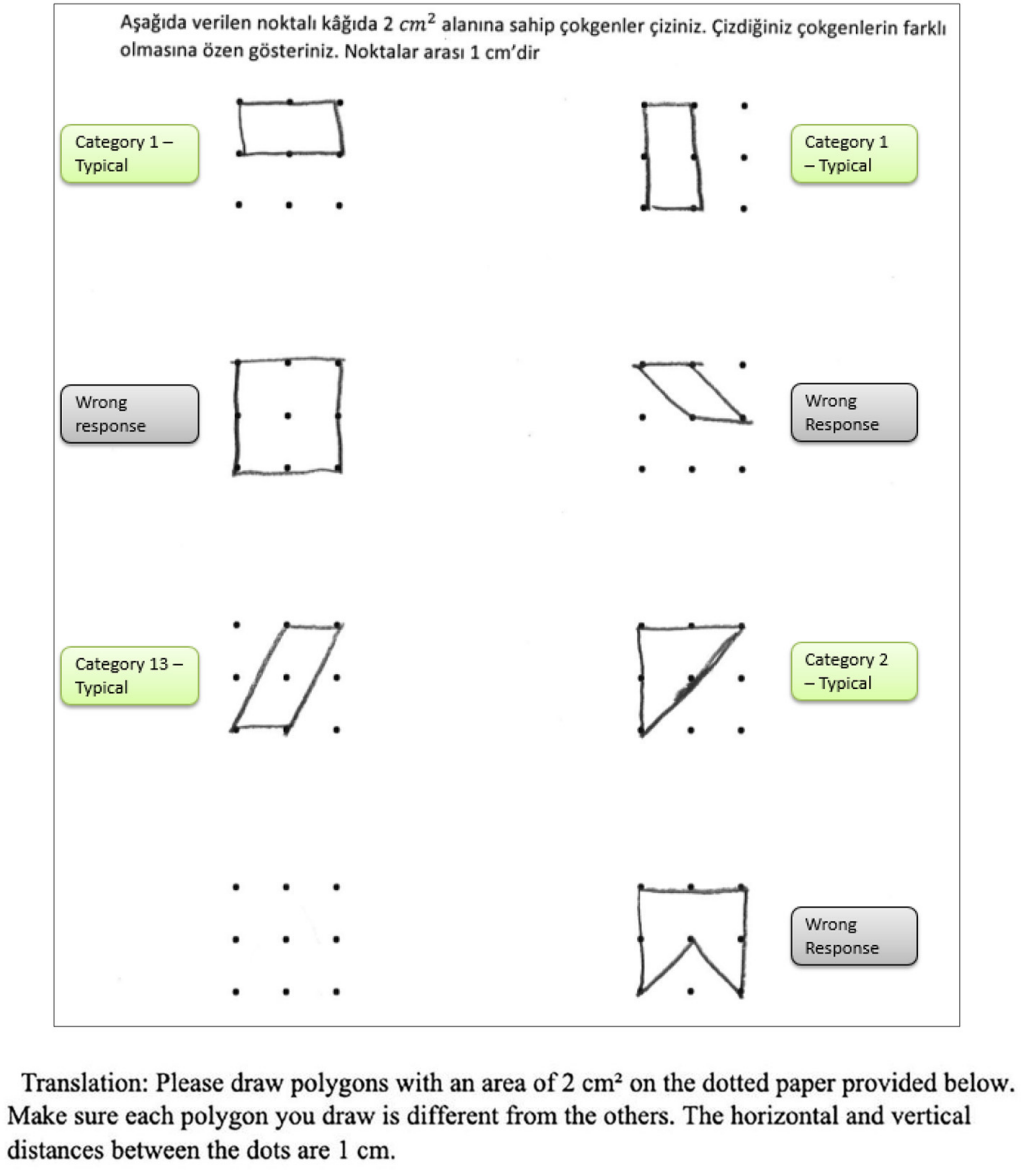
A sample student response to a DPAMPS-Task 1.

In addition to providing a sample student response to a numerical task, a sample response to a spatial task (Figure 2) is provided. The student provided seven different answers to the spatial task. Four of these responses were coded as correct responses since the area of the polygons created was 2 cm^2^ while the other polygons did not have an area of 2 cm^2^. Moreover, Responses 1 and 2 were coded in the same category since they were similar polygons (as a rotated version of each other). As a result, he received 40 points for the spatial fluency and 30 points for the spatial flexibility since there were three distinctive responses. Regarding measuring his spatial originality score, it was noticed that the most original work of the student was response 5. While only one student provided this answer, it was evaluated as a solution that appeared in less than 1 % of the sample's solutions. Therefore, he received 40 points for his spatial originality score.

### Data analysis

The first research question aims to clarify the relationship between the components of creativity in mathematical problem solving in spatial and numerical domains. To this end, data obtained from 167 participants were analyzed using SPSS-23 software. Stem-and-leaf plots and boxplots were used to evaluate the distribution of the data. As a result of the analysis, extremely high and low values were examined as possible outliers, but no problematic outliers were found (Zhao and Tan, 2016). Before examining the relationships between measurements, the normality assumption of the data set was tested. Skewness and kurtosis coefficients between−2 and +2 indicate a normal distribution (George and Mallery, 2019). The skewness and kurtosis values related to the normality of the scales are presented in detail in Table 3.

**Table 3:** Skewness and kurtosis values related to the normality of the students' scores.

**Creativity components**	**Skewness**	**Kurtosis**
Spatial fluency	0.152	−1.317
Spatial flexibility	0.708	−0.719
Spatial originality	1.562	1.784
Spatial creativity	0.540	−0.641
Numerical fluency	0.194	−0.909
Numerical flexibility	0.334	−0.678
Numerical originality	1.199	0.413
Numerical creativity	0.494	−0.486

**Figure 3:**
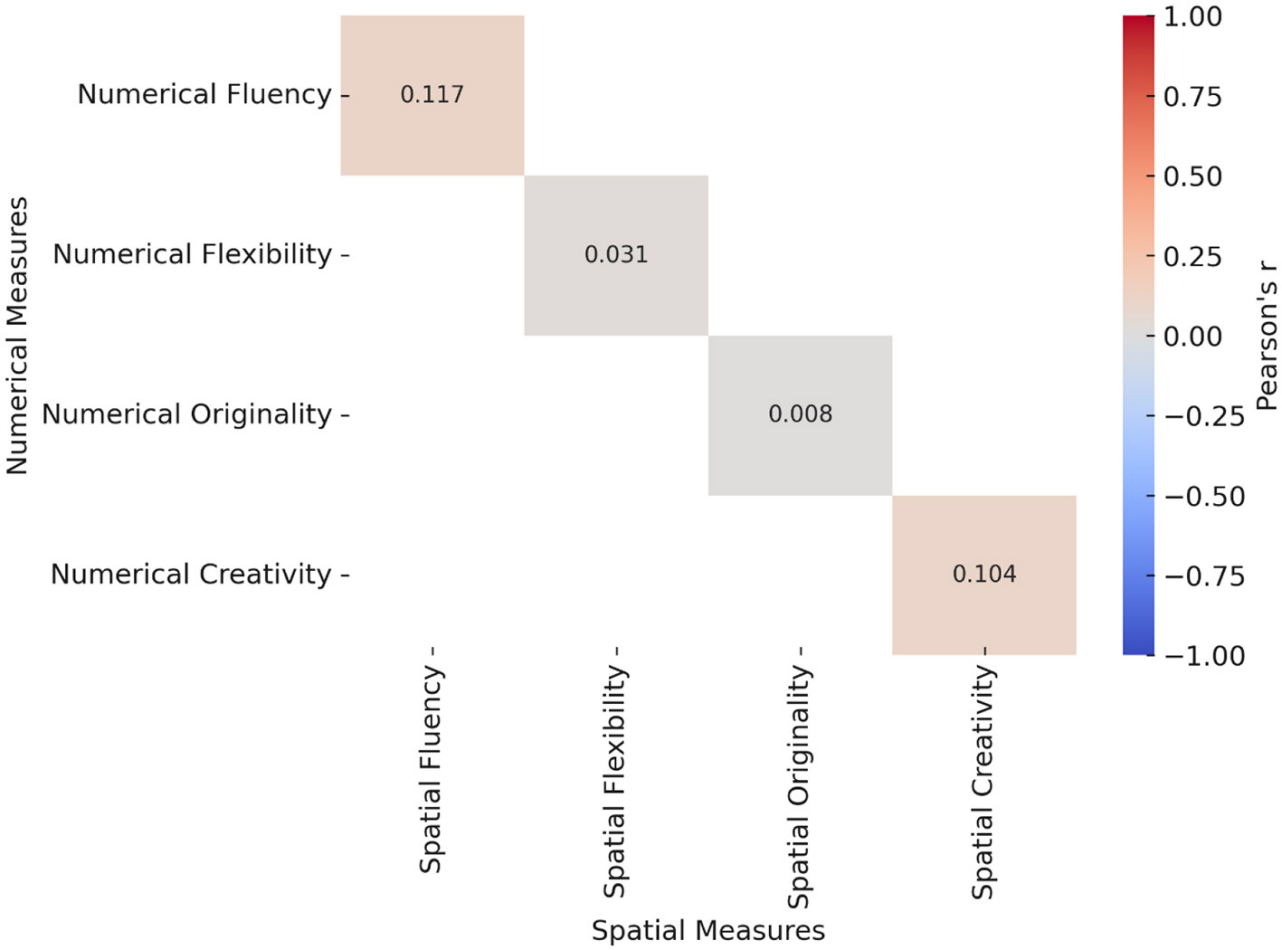
Pearson correlations between spatial and numerical creativity measures.

The second research problem aims to examine the products produced by students with different levels of creativity in numerical and spatial domain tasks. In this context, the solutions produced by students for the numerical and spatial tasks included in the DPAMPS tool were analyzed based on the product categories defined by Haylock (see Appendix 1). Moreover, the typicality of the students' products was also evaluated based on the products' alignment with the mathematics teaching program that students were exposed to and the response quality of being referred to as “prototype” concept images (Battista, 2007; Clements, 2004; Clements and Battista, 1992; Kaur, 2015; Tsamir et al., 2015). For example, a rectangle standing on a long side on a horizontal line (response 1 in Figure 3) is called a typical or prototype image (Bernabeu et al., 2021; Clements et al., 1999). Exceptionally, if sides of a polygon are not in a horizontal (or vertical) positions or it is a concave polygon, this polygon was coded as atypical because previous studies indicate that many students, prospective teachers, and in-service teachers have great difficulty in identifying polygons whose one side is not in a vertical position or concave (Bernabeu and Llinares, 2017; Bernabeu et al., 2018, 2021; De Villiers, 1994; Kaur, 2015).

Following the categorical definition of the products, students were categorized into two groups based on their creative ability scores in problem solving: high-level creativity and low-level creativity groups. This classification was determined by ranking the average total creativity scores from both tasks, from highest to lowest. Drawing from the literature (e.g., Bart et al., 2020; Renzulli, 2005), the cutoff for classification was set at the top 20%. The top 45 students with the highest creativity scores were designated as the high creative ability group (HCAG), while the remaining 122 students were categorized as the low creative ability group (LCAG). In the final stage, the similarities and differences in the categories of spatial and numerical products between the two groups were presented graphically.

## Results

### Relationship between creative ability constructs in mathematical problem solving across spatial and numerical domains

This section of the study examined the relationship between the relevant sub-dimensions of creative problem-solving processes in spatial and numerical contexts, as detailed in Figure 3. The analysis revealed no statistically significant relationship between spatial creativity and numerical creativity. Furthermore, an examination of the sub-dimensions of creativity indicated that there was no significant correlation between the variables of spatial fluency and numerical fluency, or between the variables of spatial flexibility and numerical flexibility. Similarly, the variables of spatial originality and numerical originality demonstrated no significant correlation.

### Characteristics of numerical and spatial products created by students with varying levels of creative ability

Table 4 presents the percentage distribution of product categories produced by middle school students in numerical and spatial problem-solving activities. An examination of the table reveals that in solving the numerical problem requiring the identification of a common characteristic between two natural numbers, students generated more products in the N6, N1, N7, and N2 categories. Analysis of these categories indicates that they correspond to concepts encountered in middle school education (N6) and typical responses related to factors and multiples (N2, N3), odd and even numbers (N4, N11), and the digits of a number (N7), which frequently appear in Haylock's study and are classified under typical responses. Conversely, relatively few students focused on arithmetic operations (N5, N12, N10), comparisons with other quantities (N9), digit properties (N8), visual elements (N13), and potential real-life applications (N12) in their exploration of the common characteristics of these numbers.

**Table 4:** Distribution of solution strategies used by students in numerical and spatial problem-solving activities.

**Typicality**	**Numerical categories**	**%**	**Sample product**	**Spatial categories**	**Typicality**	**%**	**Sample product**
Typical number properties	N6 (They are both numbers)	47.9	Both are natural numbers.	S1	Typical (convex) shapes	49.7	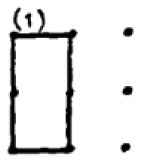
	N1 (They are both multiples of/divided by…)	46.7	Both are multiples of 4.	S2		35.9	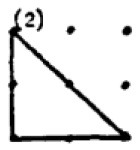
	N7 (Statements about the 6)	37.1	There are 6 in both numbers.	S3		46.7	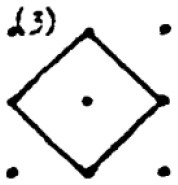
	N2 (Other statements related to multiples)	20.4	Neither is a multiple of 5.	S9		27.5	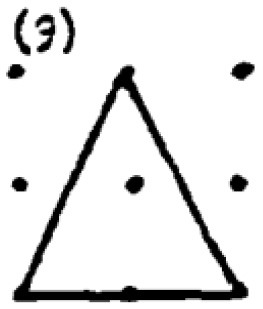
	N3 (They are both factors of…)	12.6	Both are factors of 144.	S13		0.6	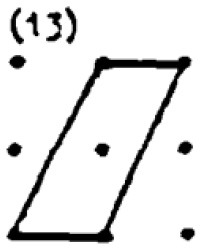
	N4 (They are both even)	11.4	These numbers are even numbers.				
	N11 (Operations on/relationships between digits)	8.4	The sum of the digits of both numbers is an odd number.	S4	Atypical (concave) shapes	1.8	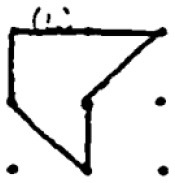
Atypical number properties	N8 (Statements about digits.)	3.0	Both have two digits.	S5		3.0	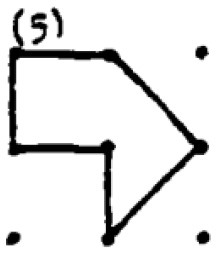
	N9 (Order properties.)	1.8	Both numbers are less than 100.	S6		3.6	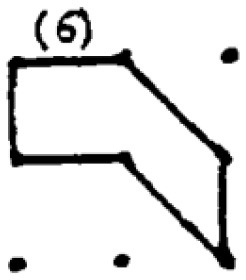
	N13 (Physical appearance)	1.8	Both numbers are in black color.	S7		4.2	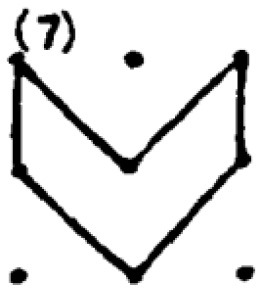
	N12 (Potential applications.)	1.2	Two numbers can also be the floor number of a building.	S8		0.6	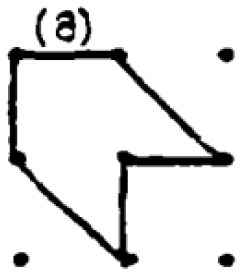
	N5 (They are both squares)	0.6	Both are perfect squares.	S10		1.8	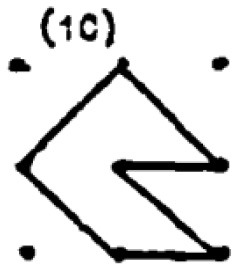
	N10 (Arithmetic relationships)	0.6	Both numbers are 6 more than a multiple of 10.	S16		0.6	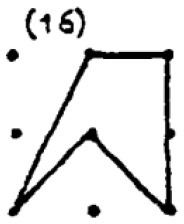

In evaluating the products created for the task of constructing a shape with an area of two square units, it is observed that students produced more items in the S1, S2, S3 and S9 categories. These products predominantly consisted of convex shapes (square, rectangle and triangle models) frequently encountered in the middle school curriculum. It is noteworthy that only a small number of students produced responses classified under the parallelogram (S13), which is also included in the typical response category. Additionally, the table shows that students' output in categories involving concave shapes is relatively low.

**Figure 4:**
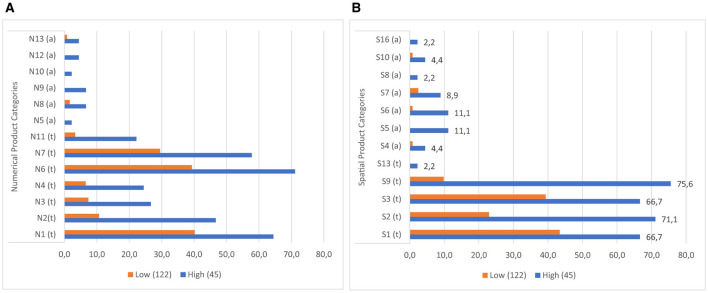
**(A)** Numerical products of students with different levels of creativity. **(B)** Spatial products of students with different levels of creativity.

The figures below illustrate the percentage distribution of products created by students at varying levels of mathematical creativity in numerical (Figure 4A) and spatial (Figure 4B) contexts. Upon analysis of the graphs, it becomes evident that HCAG students have generated a greater number of products than LCAG students across all categories of numerical and spatial contexts. This indicates that, in all numeric and spatial categories, HCAG students have produced a product, while LCAG students have not.

A detailed analysis of the products belonging to numerical contexts reveals that the products of LCAG students are generally within the typical product categories of numbers. Additionally, they demonstrate a clear focus on the digit properties of numbers (N6 and N7) and their factors (N1). It is also noteworthy that a mere fraction of LCAG students (less than 2%) were able to provide products in some of the atypical answer categories, particularly those based on arithmetic operations (N5, N9, N10, N12). In contrast, HCAG students predominantly offered responses within the conventional response categories, with a notable prevalence in the N1, N2, N6, and N7 categories. Conversely, although HCAG students were more likely to produce products in atypical categories than LCAG students, less than 10% of HCAG students were able to produce products in atypical response categories.

An evaluation of the student products in the spatial contexts reveals that, similar to the products in the numerical contexts, all students included a greater number of drawings featuring typical convex shapes, including the rectangle (S1), rhombus (S3), right triangle (S2), and triangle with a base on the ground (S9). However, the response rates of HCAG students (varying from 66% to 75%) were significantly higher than those of LCAG students (varying from 9% to 40%). Furthermore, LCAG students employed the right triangle with sides constructed on given points (S2) with greater frequency than the right triangle with sides constructed between points (S9). Notably, a considerable proportion of HCAG students constructed both types of triangles, with a greater number of students utilizing the S9 model. However, an examination of the data reveals that a mere handful of HCAG students included the typical parallelogram drawing (S13) among the convex figures, while no LCAG students included it. Furthermore, an examination of the distributions of concave drawings reveals that LCAG students did not include certain drawings (S5, S8, S13, S16), while students in both groups exhibited challenges in producing products in these categories. Conversely, it was established that HCAG students were capable of producing products in all atypical spatial categories albeit in limited quantities.

## Discussion

This study aimed to examine the relationship between creative components in spatial and numerical mathematical problem-solving tasks and to explore how students with different levels of creativity produce and categorize their mathematical outputs. Regarding the first research question that is related to the relationship between creative skills in mathematical problem-solving across spatial and numerical domains, the findings revealed no significant correlation between students' spatial and numerical creativity scores (*r* = 0.104, *p* = 0.183). This result aligns with the domain-specific perspective of creativity (Baer, 1998; Plucker and Zabelina, 2009), which suggests that creative thinking manifests differently across domains and is not necessarily transferable between contexts. While some studies have proposed an integrated view of creativity across disciplines (Plucker and Beghetto, 2004), the absence of correlation in this study supports Baer's (1998) argument for domain specificity and resonates with similar findings in mathematics education (Lin and Cho, 2011; Bicer et al., 2024a).

Studies that view creativity as a multidimensional construct suggest that domain-general and domain-specific processes may be activated together, depending on the context and task demands (Plucker and Beghetto, 2004). In mathematics education, numerical creativity is often associated with symbolic operations and algebraic reasoning, while spatial creativity is linked to mental imagery and geometric intuition (Cheng and Mix, 2014; Sternberg and Lubart, 1998). Although these two abilities are distinct, they both contribute to students‘ development of flexible and valid problem-solving strategies. Our findings show that these two types of creativity are not strongly related, but that each plays a separate role in students' success. This independence highlights the value of different creative profiles in mathematical problem solving and underscores the need for differentiated instructional design. Rather than assuming creativity develops uniformly, educators must recognize the distinct demands of each domain. Tasks that support numerical creativity might emphasize pattern recognition, arithmetic structure, or algebraic manipulation, while those promoting spatial creativity could involve dynamic geometry environments, spatial transformation challenges, or design-based modeling activities (Sorby, 2009; Wai et al., 2009). In this regard, instructional programs that offer domain-sensitive opportunities for creative engagement are essential for fostering both dimensions of mathematical thought.

Regarding the second research question, related to the characteristics of the products from students with varying mathematical creativity in numerical and spatial problems, task-based analyses have shown that students mostly resorted to traditional strategies in both contexts. One of the most striking findings of this study was that students, regardless of their creativity level, relied predominantly on routine and prototypical solution strategies in both tasks. In the numerical domain, students frequently cited basic properties such as parity or divisibility. In the spatial task, they mostly constructed standard convex shapes like rectangles and right triangles. Even among students in the high creativity group, innovative or atypical responses were rare. This pattern reveals how deeply instructional norms and curricular constraints shape students' engagement with mathematical tasks.

Research suggests that classroom practices often emphasize procedural fluency, correct answers, and algorithmic methods at the expense of flexible or divergent thinking (Lithner, 2008; Leikin and Levav-Waynberg, 2008). Vinner (1983, 1991) identified three possible interactions between a concept image in a student's mind and its definition: reshaping the image according to the definition, memorizing the definition without changing the image, or not internalizing the definition or changing the image. Experiences with examples of a concept in school, texts, or other contexts also play a role in forming a concept image. In the pedagogy of geometric concepts, instructors frequently employ a strategy of introducing a novel concept through a comprehensive explanation and a limited set of illustrative examples. This approach, as articulated by Vinner (2011), has been found to impede the establishment of a cohesive connection between the conceptual image and the definition of the concept. Unfortunately, teachers often introduce new mathematical concepts through a few prototypical examples (Vinner, 2011), which can weaken the connection between concept image and concept definition. When learners encounter only such prototypes (Hershkowitz, 1989), their judgments tend to rely on superficial visual features, leading to limited and sometimes misleading concept images. Thus, even students with high creative potential resort to typical solutions in closed-ended tasks, and curriculum materials reinforce this tendency (Houang and Schmidt, 2008; Tarr et al., 2006). To shift this tendency, pre-service and in-service teacher training programs should include explicit modules on recognizing and challenging prototype-driven reasoning (Ulusoy, 2021, 2023). Using dynamic geometry software or counter-example generation tasks can help teachers model cognitive flexibility and cultivate environments where non-routine thinking is valued (Tsamir et al., 2008; Ulusoy, 2021). Moreover, tasks that allow for open-ended and multiple solution paths are critically important (Leikin and Guberman, 2023; Vale and Barbosa, 2023). However, such tasks are insufficient represented in curriculum materials, especially at higher grade levels (Bicer et al., 2024a,b). Furthermore, teachers mostly stick to curriculum materials and do not reinterpret them for creativity-focused purposes (Bicer et al., 2021). Standardized tests and performance pressures further exacerbate these limitations, steering teachers toward a classroom culture centered on correct answers (Fleith, 2000; Robinson, 2011; Shriki, 2008). Consequently, even in open-ended tasks, students may tend to rely on internalized concept images and gravitate toward prototypical forms (Tall and Vinner, 1981; Jones, 2018).

The study also provided robust evidence that students with higher creative abilities produced a greater variety of responses in both spatial and numerical tasks, including more entries in atypical categories. This finding is consistent with literature linking mathematical creativity to greater divergence, flexibility, and originality in problem-solving (Silver, 1997; Bicer, 2021; Van Harpen and Sriraman, 2013). The top 20% of students (HCAG) demonstrated the capacity to transcend prototype images (Battista, 2007; Clements, 2004) and generate responses in underrepresented and conceptually complex categories (e.g., concave polygons or arithmetic relationships between digits). In contrast, lower creative ability students tended to produce typical, curriculum-aligned solutions, a finding that echoes previous research on the dominance of prototype-driven reasoning in school mathematics (Jones, 2018; Tsamir et al., 2015).

## Conclusions

The study found no significant correlation between students' spatial and numerical creativity scores, indicating that these two forms of creativity may function as independent cognitive resources in mathematical problem solving. While distinct, both contribute to developing flexible and valid strategies, underscoring the importance of diverse creative profiles in mathematics education. Moreover, students tended to use traditional and prototypical strategies, reflecting the influence of teaching norms and curriculum materials emphasizing procedural accuracy over creative exploration. This suggests that instructional practices and assessment pressures restrict opportunities for students to express their creative potential. To cultivate mathematical creativity, designing open-ended tasks, allowing multiple solution paths, and encouraging problem formulation, non-intuitive reasoning, and spatial exploration alongside symbolic problem solving is crucial. Breaking reliance on prototypes and routine strategies will enable students to engage with mathematics more innovatively and flexibly. Teachers can foster creativity by reinterpreting curriculum materials to invite alternative approaches, highlighting multiple solution strategies, and using counterexamples that challenge prototypical reasoning. Future research should examine the effectiveness of such creativity-focused instructional interventions in supporting diverse learner profiles and promoting more innovative engagement with mathematics.

## Limitations and future studies

This study has several limitations that should be acknowledged. First, the study assessed spatial and numerical creativity only through limited open-ended tasks. Although the tasks were selected in line with the curriculum and previous research (e.g., Bicer et al., 2020; Haylock, 1987; Kar et al., 2019; Sadak et al., 2022), single-item measures have limitations in terms of generalizability and reliability. Therefore, the findings may reflect not only the creative potential of the students but also the opportunities and limitations offered by the tasks used. Nevertheless, considering sixth-grade students' developmental characteristics and cognitive demands, an attempt was made to strike a balance between psychometric soundness and applicability. Future research should examine spatial and numerical creativity in a comprehensive and generalizable manner using a wider range of tasks. Second, the research was conducted within the Turkish educational context. Differences in textbooks, curriculum design, and teacher education practices across countries may influence the results, suggesting the need for comparative studies in diverse contexts. Third, the sample size was relatively small, and the study was carried out over a short time frame. To strengthen the evidence base, future research could employ larger participant groups and adopt longitudinal designs to trace the development of teachers' and students' concept images and definitions over time.

## Data Availability

The raw data supporting the conclusions of this article will be made available by the authors, without undue reservation.

## References

[B1] Amado N. Carreira S. Jones K. (2018). “Broadening research on mathematical problem-solving: An introduction,” in Broadening the Scope of Research on Mathematical Problem Solving: A Focus on Technology, Creativity and Affect (Cham: Springer International Publishing), 1–12.

[B2] Arikan E. E. Ünal H. (2015). Investigation of problem-solving and problem-posing abilities of seventh-grade students. Educ. Sci. Theory Pract. 15, 1403–1416. 10.12738/estp.2015.5.2678

[B3] Baer J. (1998). The case for domain specificity of creativity. Creat. Res. J. 11, 173–177. 10.1207/s15326934crj1102_7

[B4] Balka D. S. (1974). Creative ability in mathematics. Arith. Teach. 21, 633–636. 10.5951/AT.21.7.0633

[B5] Bart W. M. Can I. Hokanson B. (2020). Exploring the relation between high creativity and high achievement among 8th and 11th graders. Int. Online J. Educ. Teach. 7, 712–720.

[B6] Battista M. T. (2007). “The development of geometric and spatial thinking,” in Second Handbook of Research on Mathematics Teaching and Learning, ed. F. K. Lester (Reston, VA: NCTM-IAP), 843–908.

[B7] Beaty R. E. Seli P. Schacter D. L. (2019). Network neuroscience of creative cognition: mapping cognitive mechanisms and individual differences in the creative brain. Curr. Opin. Behav. Sci. 27, 22–30. 10.1016/j.cobeha.2018.08.013 30906824 PMC6428436

[B8] Bernabeu M. Llinares S. (2017). How do six to nine years-old children understand geometrical shapes. Educ. Matemática 29, 9–35. 10.24844/EM2902.01

[B9] Bernabeu M. Moreno M. Llinares S. (2018). “Primary school children's (9 years-old) understanding of quadrilaterals,” in Proceedings of the 42nd Conference of the International Group for the Psychology of Mathematics Education, Vol. 2, eds. E. Bergqvist, M. Österholm, C. Granberg, and L. Sumpter (Umeå: PME), 155–162.

[B10] Bernabeu M. Moreno M. Llinares S. (2021). Primary school students' understanding of polygons and the relationships between polygons. Educ. Stud. Math. 106, 251–270. 10.1007/s10649-020-10012-1

[B11] Bicer A. (2021). A systematic literature review: discipline-specific and general instructional practices fostering the mathematical creativity of students. Int. J. Educ. Math. Sci. Technol. 9, 252–281. 10.46328/ijemst.1254

[B12] Bicer A. Chamberlin S. A. Matute K. Jackson T. Krall G. (2024a). The relationship between pre-service teachers' spatial thinking ability and their mathematical creativity in the context of problem posing. Res. Math. Educ. 26, 544–568. 10.1080/14794802.2023.2201619

[B13] Bicer A. Lee Y. Perihan C. Capraro M. M. Capraro R. M. (2020). Considering mathematical creative self-efficacy with problem posing as a measure of mathematical creativity. Educ. Stud. Math. 105, 457–485. 10.1007/s10649-020-09995-8

[B14] Bicer A. Marquez A. Colindres K. V. M. Schanke A. A. Castellon L. B. Audette L. M.. (2021). Investigating creativity-directed tasks in middle school mathematics curricula. Think. Skills Creat. 40:100823. 10.1016/j.tsc.2021.100823

[B15] Bicer A. Aleksani H. Butler C. Jackson T. Smith T. D. Bostick M. (2024b). Mathematical creativity in upper elementary school mathematics curricula. Think. Skills Creat. 51:101462. 10.1016/j.tsc.2024.101462

[B16] Chamberlin S. A. Moon S. M. (2005). Model-eliciting activities as a tool to develop and identify creatively gifted mathematicians. J. Second. Gift. Educ. 17, 37–47. 10.4219/jsge-2005-393

[B17] Cheng Y.-L. Mix K. S. (2014). Spatial training improves children's mathematics ability. J. Cogn. Dev. 15, 2–11. 10.1080/15248372.2012.725186

[B18] Chrysikou E. G. (2019). Creativity in and out of (cognitive) control. Curr. Opin. Behav. Sci. 27, 94–99. 10.1016/j.cobeha.2018.09.014

[B19] Chrysikou E. G. Thompson-Schill S. L. (2011). Dissociable brain states linked to common and creative object use. Hum. Brain Mapp. 32, 665–675. 10.1002/hbm.21056 20533561 PMC3846690

[B20] Clark G. Zimmerman E. (1997). The influence of theoretical frameworks on Clark and Zimmerman's research about art talent development. J. Aesthet. Educ. 31, 49–63. 10.2307/3333143

[B21] Clements D. H. (2004). “Geometric and spatial thinking in early childhood education,” in Engaging Young Children in Mathematics: Standards for Early Childhood Mathematics Education, ed. D. H. Clements (Lawrence Erlbaum Associates), 267–297.

[B22] Clements D. H. Battista M. T. (1992). “Geometry and spatial reasoning,” in Handbook of Research on Mathematics Teaching and Learning, ed. D. A. Grouws (New York, NY: MacMillan), 420–464.

[B23] Clements D. H. Swaminathan S. Hannibal M. A. Z. Sarama J. (1999). Young children's concepts of shape. J. Res. Math. Educ. 30, 192–212. 10.2307/749610

[B24] De Villiers M. (1994). The role and function of a hierarchical classification of quadrilaterals. Learn. Math. 14, 11–18.

[B25] Di Martino P. Zan R. (2010). “Me and maths”: towards a definition of attitude grounded on students' narratives. J. Math. Teach. Educ. 13, 27–48. 10.1007/s10857-009-9134-z

[B26] Ev-Çimen E. E. Yıldız S. (2017). An investigation of problem posing activities in secondary school mathematics textbooks. Turk. J. Comput. Math. Educ. 8, 378–407.

[B27] Fleith S. D. (2000). Teacher and student perceptions of creativity in the classroom environment. Roeper Rev. 22, 148–153. 10.1080/02783190009554022

[B28] Gardner H. (1988a). “12 Creative lives and creative works: A synthetic scientific approach,” in The Nature of Creativity: Contemporary Psychological Perspectives, ed. R. J. Sternberg (New York, NY: Cambridge University Press),298–324.

[B29] Gardner H. (1988b). Creativity: An interdisciplinary perspective. Creat. Res. J. 1, 8–26. 10.1080/10400418809534284

[B30] George D. Mallery P. (2019). IBM SPSS Statistics 26 Step by Step: A Simple Guide and Reference. Milton Park: Routledge.

[B31] Grégoire J. (2016). Understanding creativity in mathematics for improving mathematical education. J. Cogn. Educ. Psychol. 15, 24–36. 10.1891/1945-8959.15.1.24

[B32] Guilford J. P. (1959). Three faces of intellect. Am. Psychol. 14, 469–479. 10.1037/h0046827

[B33] Hannula M. S. (2002). Attitude towards mathematics: emotions, expectations, and values. Educ. Stud. Math. 49, 25–46. 10.1023/A:1016048823497

[B34] Haylock D. (1985). Conflicts in the assessment and encouragement of mathematical creativity in schoolchildren. Int. J. Math. Educ. Sci. Technol. 16, 547–553. 10.1080/0020739850160412

[B35] Haylock D. (1987). A framework for assessing mathematical creativity in school children. Educ. Stud. Math. 18, 59–74. 10.1007/BF00367914

[B36] Haylock D. (1997). Recognizing mathematical creativity in schoolchildren. ZDM 29, 68–74. 10.1007/s11858-997-0002-y

[B37] Haylock D. W. (1984). Aspects of Mathematical Creativity in Children Aged 11–12 (Doctoral dissertation). London: University of London.

[B38] Hershkowitz R. (1989). Visualization in geometry: two sides of the coin. Focus Learn. Probl. Math. 11, 61–76.

[B39] Houang R. T. Schmidt W. H. (2008). “TIMSS international curriculum analysis and measuring educational opportunities,” in 3rd IEA International Research Conference (Taipei, Chinese Taipei). Available online at: https://www.researchgate.net/profile/Richard-Houang/publication/268395948_TIMSS_International_Curriculum_Analysis_and_Measuring_Educational_Opportunities/links/54cfd7970cf29ca811006390/TIMSS-International-Curriculum-Analysis-and-Measuring-Educational-Opportunities.pdf (Accessed October 8, 2025).

[B40] Humphreys L. G. Lubinski D. Yao G. (1993). Utility of predicting group membership and the role of spatial visualization in becoming an engineer, physical scientist, or artist. J. Appl. Psychol. 78:250. 10.1037//0021-9010.78.2.250 8482696

[B41] Jones S. R. (2018). Prototype images in mathematics education: the case of the graphical representation of the definite integral. Educ. Stud. Math. 97, 215–234. 10.1007/s10649-017-9794-z

[B42] Kar T. Özdemir E. Öçal M. F. Güler G. Ipek A. S. (2019). “Indicators of prospective mathematics teachers' success in problem solving: the case of creativity in problem posing,” in Proceedings of the 43rd Conference of the International Group for the Psychology of Mathematics Education, Vol. 2, 456–463.

[B43] Kattou M. Kontoyianni K. Pitta-Pantazi D. Christou C. (2013). Connecting mathematical creativity to mathematical ability. ZDM 45, 167–181. 10.1007/s11858-012-0467-1

[B44] Kaur H. (2015). Two aspects of young children's thinking about different types of dynamic triangles: prototypicality and inclusion. ZDM Math. Educ. 47, 407–420. 10.1007/s11858-014-0658-z

[B45] Kell H. J. Lubinski D. Benbow C. P. Steiger J. H. (2013). Creativity and technical innovation: spatial ability's unique role. Psychol. Sci. 24, 1831–1836. 10.1177/0956797613478615 23846718

[B46] Khalid M. Saad S. Abdul Hamid S. R. Ridhuan Abdullah M. Ibrahim H. Shahrill M. (2020). Enhancing creativity and problem solving skills through creative problem solving in teaching mathematics. Creat. Stud. 13, 270–291. 10.3846/cs.2020.11027

[B47] Kim H. Cho S. Ahn D. (2003). Development of mathematical creative problem solving ability test for identification of the gifted in math. Gift. Educ. Int. 18, 164–174. 10.1177/026142940301800206

[B48] Kim S. Choe I. Kaufman J. C. (2019). The development and evaluation of the effect of creative problem-solving program on young children's creativity and character. Think. Skills Creat. 33:100590. 10.1016/j.tsc.2019.100590

[B49] Lee S. H. Hoffman K. D. (2014). The “Iron Inventor”: using creative problem solving to spur student creativity. Mark. Educ. Rev. 24, 69–74. 10.2753/MER1052-8008240112

[B50] Lee-Post A. (2019). Developing numeracy and problem-solving skills by overcoming learning bottlenecks. J. Appl. Res. High. Educ. 11, 398–414. 10.1108/JARHE-03-2018-0049

[B51] Leikin R. (2007). “Habits of mind associated with advanced mathematical thinking and solution spaces of mathematical tasks,” in Proceedings of the Fifth Conference of the European Society for Research in Mathematics Education, eds. D. Pitta-Pantazi, and G. Philippou, 2330–2339.

[B52] Leikin R. (2009). “Exploring mathematical creativity using multiple solution tasks,” in Creativity in Mathematics and the Education of Gifted Students, eds. R. Leikin, A. Berman, and B. Koichu (Sense Publishers), 129–145.

[B53] Leikin R. Berman A. Koichu B. (2009). Creativity in Mathematics and the Education of Gifted Students. Rotterdam, The Netherlands: Sense.

[B54] Leikin R. Guberman R. (2023). “Creativity and challenge: tasks complexity as a function of insight and multiplicity solutions,” in Mathematical Challenges for All. Research in Mathematics Education, ed. R. Leikin (Springer), 325–342. 10.1007/978-3-031-18868-8

[B55] Leikin R. Levav-Waynberg A. (2008). Solution spaces of multiple solution connecting tasks as a mirror of the development of mathematics teachers' knowledge. Can. J. Sci. Math. Technol. Educ. 8, 233–251. 10.1080/14926150802304464

[B56] Levav-Waynberg A. Leikin R. (2012). The role of multiple solution tasks in developing knowledge and creativity in geometry. J. Math. Behav. 31, 73–90. 10.1016/j.jmathb.2011.11.001

[B57] Lin C. Y. Cho S. (2011). Predicting creative problem solving in math from a dynamic system model of creative problem solving ability. Creat. Res. J. 23, 255–261. 10.1080/10400419.2011.595986

[B58] Lithner J. (2008). A research framework for creative and imitative reasoning. Educ. Stud. Math. 67, 255–276. 10.1007/s10649-007-9104-2

[B59] Liu L. M. (2007). The relationships between creativity, drawing ability, and visual/spatial intelligence: a study of Taiwan's third-grade children. Asia Pac. Educ. Rev. 8, 343–352. 10.1007/BF03026464

[B60] Lohman D. F. (1996). “Spatial ability and g,” in Human Abilities: Their Nature and Measurement, eds. I. Dennis, and P. Tapsfield (Lawrence Erlbaum Associates), 97–116.

[B61] Martin A. J. Marsh H. W. (2008). Academic buoyancy: towards an understanding of students' everyday academic resilience. J. Sch. Psychol. 46, 53–83. 10.1016/j.jsp.2007.01.002 19083351

[B62] Millett A. Askew M. Brown M. (2004). The impact of the national numeracy strategy in Year 4 (II): teaching. Res. Math. Educ. 6, 191–205. 10.1080/14794800008520137

[B63] Ministry of National Education (1949). Ortaokul Programi (Middle School Curriculum). Ankara: MEB. Turkish.

[B64] Ministry of National Education (2024). Ortaokul Matematik Dersi Ögretim Programi (5, 6, 7 ve 8. Siniflar) [Middle School Mathematics Teaching Program (Grades 5, 6, 7 and 8)]. Ankara: MEB. Turkish.

[B65] Murphy K. R. Davidshofer C. O. (2001). Psychological Testing. Upper Saddle River, NJ: Prentice Hall.

[B66] Plucker J. Zabelina D. (2009). Creativity and interdisciplinarity: one creativity or many creativities? ZDM 41, 5–11. 10.1007/s11858-008-0155-3

[B67] Plucker J. A. Beghetto R. A. (2004). “Why creativity is domain general, why it looks domain specific, and why the distinction does not matter,” in Creativity: From Potential to Realization, eds. R. J. Sternberg, E. L. Grigorenko, and J. L. Singer (American Psychological Association), 153–167.

[B68] Plucker J. A. Qian M. Wang S. (2011). Is originality in the eye of the beholder? Comparison of scoring techniques in the assessment of divergent thinking. J. Creat. Behav. 45, 1–22. 10.1002/j.2162-6057.2011.tb01081.x

[B69] Pugalee D. K. (1999). Constructing a model of mathematical literacy. Clear. House 73, 19–22. 10.1080/00098659909599632

[B70] Purnomo H. Sa'dijah C. Permadi H. Anwar L. Cahyowati E. T. D. (2023). “Mathematical creative processing abilities of junior high school students' in numeracy tasks,” in AIP Conference Proceedings, Vol. 2569 (Melville, NY: AIP Publishing).

[B71] Reiter-Palmon R. Forthmann B. Barbot B. (2019). Scoring divergent thinking tests: a review and systematic framework. Psychol. Aesthet. Creat. Arts 13:144. 10.1037/aca0000227

[B72] Renzulli J. S. (2005). Applying gifted education pedagogy to total talent development for all students. Theory Pract. 44, 80–89. 10.1207/s15430421tip4402_2

[B73] Robinson K. (2011). Out of Our Minds: Learning to Be Creative. West Sussex: Capstone.

[B74] Runco M. A. Jaeger G. J. (2012). The standard definition of creativity. Creat. Res. J. 24, 92–96. 10.1080/10400419.2012.650092

[B75] Sadak M. Incikabi L. Ulusoy F. Pektas M. (2022). Investigating mathematical creativity through the connection between creative abilities in problem posing and problem solving. Think. Skills Creat. 45:101108. 10.1016/j.tsc.2022.101108

[B76] Shepard R. N. Metzler J. (1971). Mental rotation of three-dimensional objects. Science 171, 701–703. 10.1126/science.171.3972.701 5540314

[B77] Shriki A. (2008). “Towards promoting creativity in mathematics of pre-service teachers: The case of creating a definition,” in Proceedings of the 5th International Conference on Creativity in Mathematics and the Education of Gifted Students, ed. R. Leikin (Haifa), 201–210.

[B78] Silver E. A. (1997). Fostering creativity through instruction rich in mathematical problem solving and problem posing. ZDM 29, 75–80. 10.1007/s11858-997-0003-x

[B79] Sorby S. A. (2009). Educational research in developing 3-D spatial skills for engineering students. Int. J. Sci. Educ. 31, 459–480. 10.1080/09500690802595839

[B80] Sriraman B. (2009). The characteristics of mathematical creativity. ZDM 41, 13–27. 10.1007/s11858-008-0114-z

[B81] Steen L. A. (2002). Quantitative literacy: why numeracy matters for schools and colleges. Focus 22, 8–9.

[B82] Sternberg R. J. (1997). Thinking Styles. Cambridge: Cambridge University Press.

[B83] Sternberg R. J. Lubart T. I. (1998). “The concept of creativity: Prospects and paradigms,” in Handbook of Creativity, ed. R. J. Sternberg (Cambridge: Cambridge University Press), 3–15.

[B84] Stoltz P. G. (1999). Adversity Quotient: Turning Obstacles into Opportunities. Hoboken, NJ: John Wiley and Sons.

[B85] Tall D. Vinner S. (1981). Concept image and concept definition in mathematics with particular reference to limits and continuity. Educ. Stud. Math. 12, 151–169. 10.1007/BF00305619

[B86] Tarr J. E. Chávez Ó, Reys, R. E. Reys B. J. (2006). From the written to the enacted curricula: the intermediary role of middle school mathematics teachers in shaping students' opportunity to learn. Sch. Sci. Math. 106, 191–201. 10.1111/j.1949-8594.2006.tb18075.x

[B87] Titus P. A. Koppitsch S. (2018). Exploring business students' creative problem-solving preferences. J. Educ. Bus. 93, 242–251. 10.1080/08832323.2018.1465021

[B88] Torrance E. P. (1966). The Torrance Tests of Creative Thinking: Norms-Technical Manual. Research Edition. Verbal Tests, Forms A and B. Figural Tests, Forms A and B. Princeton, NJ: Personal Press.

[B89] Torrance E. P. (1988). “The nature of creativity as manifest in its testing,” in The Nature of Creativity: Contemporary Psychological Perspectives, ed. R. J. Sternberg (Cambridge: Cambridge University Press), 43–75.

[B90] Tsamir P. Tirosh D. Levenson E. (2008). Intuitive non examples: the case of triangles. Educ. Stud. Math. 69, 81–95. 10.1007/s10649-008-9133-5

[B91] Tsamir P. Tirosh D. Levenson E. Barbakai R. Tabach M. (2015). Early-years teachers' concept images and concept definitions: triangles, circles, and cylinders. ZDM Math. Educ. 47, 497–509. 10.1007/s11858-014-0641-8

[B92] Tyagi T. K. (2016). Is there a causal relation between mathematical creativity and mathematical problem solving performance? Int. J. Math. Educ. Sci. Technol. 47, 388–394. 10.1080/0020739X.2015.1075612

[B93] Ulusoy F. (2021). Prospective early childhood and elementary school mathematics teachers' concept images and concept definitions of triangles. Int. J. Sci. Math. Educ. 19, 1057–1078. 10.1007/s10763-020-10105-6

[B94] Ulusoy F. (2023). Middle school students' reasoning with regards to parallelism and perpendicularity of line segments. Int. J. Math. Educ. Sci. Technol. 54, 1187–1206. 10.1080/0020739X.2022.2049384

[B95] Ulusoy F. Incikabi L. (2020). Middle school teachers' use of compulsory textbooks in instruction of mathematics. Int. J. Math. Teach. Learn. 21, 1–18. 10.4256/ijmtl.v21i1.227

[B96] Ulusoy F. Incikabi L. (2023). Preservice mathematics teachers' selection of curriculum resources in individual and group lesson planning processes. Int. J. Math. Educ. Sci. Technol. 54, 557–578. 10.1080/0020739X.2021.1958944

[B97] Ulusoy F. Sadak M. Incikabi L. Pektas M. (2025). Exploring gender differences in mathematical creativity: linking problem posing and problem solving. Psychol. Sch. 62, 4327–4343. 10.1002/pits.70060

[B98] Uttal D. H. Meadow N. G. Tipton E. Hand L. L. Alden A. R.. (2013). The malleability of spatial skills: a meta-analysis of training studies. Psychol. Bull. 139, 352–402. 10.1037/a0028446 22663761

[B99] Vale I. Barbosa A. (2023). “Visualization: a pathway to mathematical challenging tasks,” in Mathematical Challenges for All. Research in Mathematics Education, ed. R. Leikin (Springer), 283–306.

[B100] Valentine A. Belski I. Hamilton M. (2017). Developing creativity and problem-solving skills of engineering students: a comparison of web-and pen-and-paper-based approaches. Eur. J. Eng. Educ. 42, 1309–1329. 10.1080/03043797.2017.1291584

[B101] Van Harpen X. Y. Sriraman B. (2013). Creativity and mathematical problem posing: an analysis of high school students' mathematical problem posing in China and the USA. Educ. Stud. Math. 82, 201–221. 10.1007/s10649-012-9419-5

[B102] Vinner S. (1983). Concept definition, concept image and the notion of function. Int. J. Math. Educ. Sci. Technol. 14, 293–305. 10.1080/0020739830140305

[B103] Vinner S. (1991). “The role of definitions in the teaching and learning of mathematics,” in Advanced Mathematical Thinking, ed. D. Tall (Dordrecht, The Netherlands: Kluwer), 65–81.

[B104] Vinner S. (2011). The role of examples in the learning of mathematics and in everyday thought processes. ZDM 43, 247–256. 10.1007/s11858-010-0304-3

[B105] Wai J. Lubinski D. Benbow C. P. (2009). Spatial ability for STEM domains: aligning over 50 years of cumulative psychological knowledge solidifies its importance. J. Educ. Psychol. 101, 817–835. 10.1037/a0016127

[B106] Witkin H. A. Goodenough D. R. Karp S. A. (1967). Stability of cognitive style from childhood to young adulthood. J. Pers. Soc. Psychol. 7, 291–300. 10.1037/h0025070 6065857

[B107] Yana S. I. Panglipur I. R. Anas A. (2024). Analysis of students' creativity in solving numeracy literacy problems on higher order thinking skills (HOTS) material on lines and angles. J. Educ. Learn. Math. Res. 5, 104–114. 10.37303/jelmar.v5i2.156

[B108] Yuan X. Sriraman B. (2011). “An exploratory study of relationships between students' creativity and mathematical problem posing abilities—comparing Chinese and U.S students,” in The Elements of Creativity and Giftedness in Mathematics, eds. B. Sriraman, and K. Lee (Rotterdam, The Netherlands: Sense Publishers), 5–28.

[B109] Zhao R. Tan V. Y. (2016). Online nonnegative matrix factorization with outliers. IEEE Trans. Signal Process. 65, 555–570. 10.1109/TSP.2016.2620967

