# Supplementary material for: Investigating middle school students' creative problem solving in numerical and spatial domains
Source: Front Psychol. 2025 Nov 4;16:1686498. doi: 10.3389/fpsyg.2025.1686498 (PMC12624508; doi:10.3389/fpsyg.2025.1686498)
Supplement: Supplementary file 1 [file Supplementary_file_1.docx]

**Appendix 1. Response categories for the DPAMPS tasks**


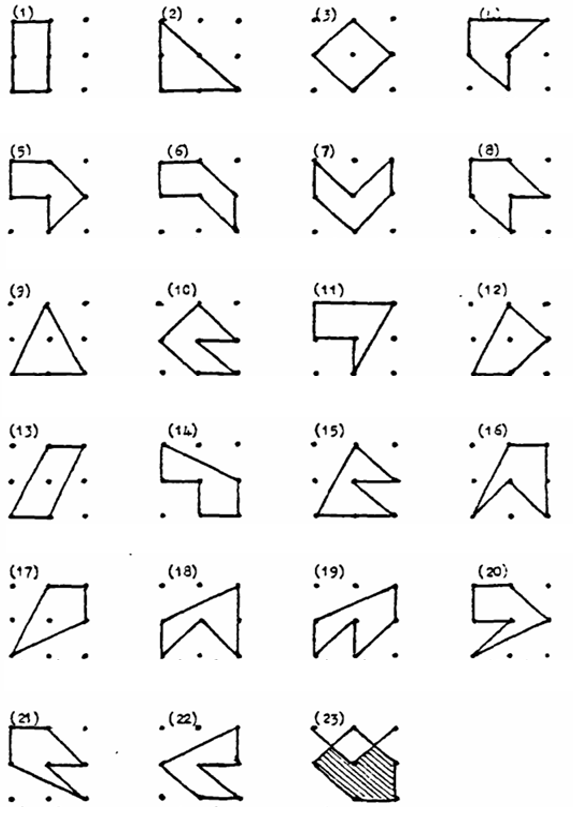


Figure 1. Nine-dot areas response categories (Haylock, 1984, p. 149)

Typical polygons: 1, 2, 3, 9, 12, 13, 17

Atypical polygons: 4, 5, 6, 7, 8, 10, 11, 14, 15, 16, 18, 19, 20, 21, 22, 23

Table 1. Acceptable response categories for numerical task (Haylock,1984, p. 181)

| Response categories | | Typicality |
| --- | --- | --- |
| #1 | They are both multiples of... (or, both can be divided by…) | Typical |
|  | a) 8  b) 4  c) 2  d) 1  e) 0.5, ½  f) An even number  g) Themselves |  |
| #2 | Other statements related to multiples | Typical |
|  | a) They are both not multiples of 5, 10, etc. |  |
| #3 | They are both factors of.. | Typical |
|  | a) They are both factors of 144  b) They are both factors of 576  c) They are both not factors of 100 |  |
| #4 | They are both even | Typical |
| #5 | They are both squares | Atypical |
| #6 | They are both numbers | Typical |
| #7 | Statements about the 6 | Typical |
|  | a) They both end in 6, have 6 units  b) They both contain a 6 |  |
| #8 | Various statements about digits. They both have: | Atypical |
|  | a) An even digit, an odd digit, one odd and one even digit, first digit odd, second digit even  b) Two digits, tens and units, a tens digit and a units digit  c) Not a single digit  d) Two different digits  e) Last digit divisible by 3, 2, 1 (first digit divisible by 1)  f) First digit prime (accepted although strictly 1 is not prime)  g) They are both in the sixties when digits reversed  h) They both begin with 9 when inverted |  |
| #9 | Order properties. | Atypical |
|  | a) Greater than some integer  b) Less than some integer  c) Between two integers  d) Greater than 15½  e) Greater than every number less than 16 |  |
| #10 | Arithmetic Relationships. They both: | Atypical |
|  | a) Are 4 less than a multiple of 10 (are rounded up to nearest 10 if 4 added), or 5  b) Are 6 more than a multiple of 10  c) Give remainder when divided by 10, give remainder 6 when divided by 10  d) Give remainder 1 when divided by 5  e) Will end in 8 when multiplied by 3  f) Will end in 8 when halved |  |
| #11 | Operations on/relationships between digits | Typical |
|  | a) They both have the first digit a factor of the second  b) They both have the sum of their digits less than 10  c) They both have the sum of their digits odd |  |
| #12 | Potential applications | Atypical |
|  | a) They both can be percentages  b) They both can be door numbers |  |
| #13 | Physical appearance | Atypical |
|  | a) In a box, square  b) In same size box  c) Same size, height  d) In black ink  e) On white background  f) Not pink, not on green background  g) Away from edges of the box  h) Same way up  i) Written clearly  j) On this paper  k) Part of a question, test  l) Being looked at by the class  m) Surrounded by words  n) They both contain curves, loops |  |
